# Rehabilitation of a complex midfacial defect by means of a zygoma-implant-supported prosthesis and nasal epithesis: a novel technique

**DOI:** 10.1186/s40729-016-0043-5

**Published:** 2016-04-01

**Authors:** Lorenzo Trevisiol, Pasquale Procacci, Antonio D’Agostino, Francesca Ferrari, Daniele De Santis, Pier Francesco Nocini

**Affiliations:** Department of Surgery, Section of Oral and Maxillofacial Surgery, University of Verona, Policlinico “Giovanni Battista Rossi”, Piazzale Ludovico Antonio Scuro, 10, 37134 Verona, Italy

**Keywords:** Extraoral rehabilitation, Obturator prosthesis, Rhinectomy, Zygoma implants, Maxillectomy

## Abstract

**Purpose:**

Several authors have described zygoma implants as a reliable surgical option to rehabilitate severe maxillary defects in case of extreme atrophy or oncological resections. The aim of this study is to report a new technical approach to the rehabilitation of a complex oronasal defect by means of a zygoma-implant-supported full-arch dental prosthesis combined with a nasal epithesis.

**Patients and methods:**

The patient presented with a subtotal bilateral maxillectomy and total rhinectomy defect because of a squamous cell carcinoma of the nose. No reconstructive surgery was performed because of the high risk of recurrence; moreover, the patient refused any secondary procedure. After surgery, the patient presented a wide palatal defect associated to the absence of the nasal pyramid. Zygoma-retained prostheses are well documented, and they offer good anchorage in rehabilitating wide defects after oncological surgery and a good chance for patients to improve their quality of life. We hereby describe two prosthetic devices rehabilitating two iatrogenic defects by means of a single intraoral implant-supported bar extending throughout the oronasal communication, thus offering nasal epithesis anchorage.

**Results:**

At 1-year follow-up after functional prosthetic loading, no implant failure has been reported. Clinical and radiological follow-up showed no sign of nasal infection or peri-implantitis. The patient reported a sensitive improvement of his quality of life.

**Conclusions:**

Simultaneous oral and nasal rehabilitation of complex oronasal defects with zygoma-implant-supported dental prosthesis and nasal epithesis represents a reliable surgical technique. According to this clinical report, the above-mentioned technique seems to be a valuable treatment option as it is safe, reliable and easy to handle for both surgeon and patient.

## Background

The use of zygoma implants in the rehabilitation of patients who underwent surgical resection for oral cancer has been widely described [1–3]. There are several possibilities that can be considered when evaluating the possibility of surgical reconstruction after the first cancer resection, such as microvascular free flaps or rotation flaps, but it is sometimes necessary to monitor the healing process and the defect site in order to readily detect recurrences that may occur in high-risk patients [4, 5]. While dealing with facial defects, it is mandatory to consider that this kind of defect has a big impact on the patient’s quality of life [6, 7]. For this reason, medical science made a strong effort in developing rehabilitation solutions that enable operated patients to re-achieve a normal life as soon as possible. According to this objective, zygoma implants allow to reconstruct full arch even in case of conspicuous bone defects with no indication to grafting procedures [6, 8]. Furthermore, in case of wide midfacial resections with oronasal communication, zygoma implants may be used through the communication to support an extraoral nasal prosthesis. This article describes the rehabilitation of two defects, one intraoral and one extraoral, resulting from a single surgical act. Both intra- and extraoral prosthetic rehabilitation are supported by four zygoma implants positioned in the resected maxilla in order to create an artificial nose and a prosthetic denture.

## Case presentation

### Materials and methods

The patient, a male 46 years old at the time of our visit, underwent surgical resection of nasal pyramid and premaxilla including the whole upper jaw teeth sparing nasal bones. When the patient came to our clinic, apart from the defect resulting from the resection, he presented with a retraction scar crossing the upper lip from the floor of the nasal defect through the filtrum. The surgical resection was performed in another clinic the previous year, and since then, the patient experienced a severe decrease in the quality of social life including the loss of job and falling into reactive depression. The histological aspect of the neoplasia was characterized by high malignancy and contraindicated a microvascular flap reconstruction in order to allow the inspection of the nasal cavity and the facial skin nearby the nasal defect during follow-up appointments.

Furthermore, the conspicuous defect and the different kind of tissues needed would have required multiple donor sites, making the achievement of a good aesthetic and functional result quite challenging. Insofar, due to the entity of the defect, the uncertain outcome of the surgical reconstruction, time-costing evaluation and follow-up need, the patient was proposed to undergo zygoma-implant-supported prosthetic restorations.

#### Surgical treatment

Radiographic examination was carried out by means of CT scans of the maxillofacial complex. After the evaluation of the residual maxillary bone, insertion of four zygoma implants was planned.

**Fig. 1:**
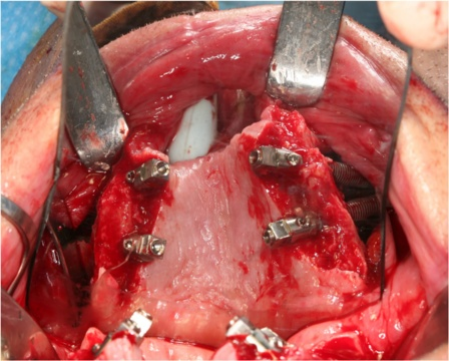
Intraoperative view of the zygoma implants placed in the residual maxilla

The surgical intervention was performed under general anaesthesia. Our surgical treatment started with an incision extended from the palatal aspect of the second molar site to the crestal aspect of the canine site bilaterally, with two posterior release incisions. A full-thickness flap was then elevated, and the anterolateral wall of the maxilla was exposed. An oval-shaped window was first drawn and was then opened trough the upper aspect of the maxillary buttress using a large round diamond bur. These windows are used to check the right direction of the zygomatic fixtures during their insertion trough the zygomatic bone. Once the maxillary buttress has been prepared bilaterally, the zygoma implant insertion could start. The preoperative planning provided the insertion of four zygomatic fixtures (Branemark System Zygoma, Zygoma TiUnite® Implant, Nobel Biocare, Goteborg, Sweden), one through the first molar area and one through the lateral canine area on both sides (Fig. 1). The reflected mucoperiosteal flap was then sutured with resorbable suture (Polysorb 4.0, Covidien, Mansfield, MA, USA).

Cortical steroids were administered for the first two postoperative days. A postoperative 10-day cycle of antibiotic therapy (amoxicillin 1000 mg TID) was administered. Analgesics were administered as required. Sutures were removed 15 days after surgery. A soft diet was recommended for the first 2 weeks.

**Fig. 2:**
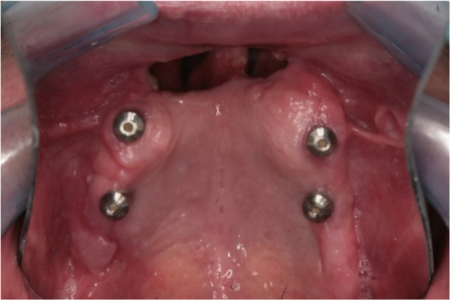
The healing abutments positioned onto fixtures and the oronasal communication

Three months afterwards, healing abutments were connected (Fig. 2) [4].

#### Prosthodontic treatment

Approximately 4 weeks after healing abutment connection, intraoral defect including implant abutment and extraoral paranasal defect impressions were taken. The technician managed two different casts: one cast for nasal wax up and one cast for dental wax up. Superior implant bar supported by [4] zygoma implants was designed crossing the palatal defect in order to manufacture palatal obturator at a second time. Furthermore, two metal abutments were lodged and fused on the cranial surface of the bar in order to receive epithesis attachments. The abutments acted as primary crowns and secondary crowns, press-fitted on abutments and were used to take an extraoral position impression of the abutments using the nasal wax up as an individual. In this way, the technician could connect OTK (Ball abutment) attachments on the internal surface of the epithesis and thanks to secondary crowns, the nasal prosthesis can be removed for prosthetic aftercare and follow-up inspections. OTK attachments are commonly used because of their retention in overdenture prosthetic rehabilitation. The female part of this peculiar type of ball attachment is made out of Teflon™ (politetrafluoroetilene) while the male part consisted of a titanium structure. A complete implant-supported bar with two bolt prosthesis was made in order to provide superior arch rehabilitation. At the time of delivery, the palatal defect was closed by a soft base material. The nasal epithesis was made of silicone with an acrylic resin internal plate hosting female OTK attachments, whereas male parts were on the secondary crowns.

### Results

**Fig. 3:**
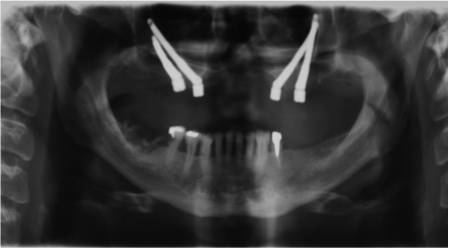
Postoperative panorex showing the symmetric distribution of the fixtures

**Fig. 4:**
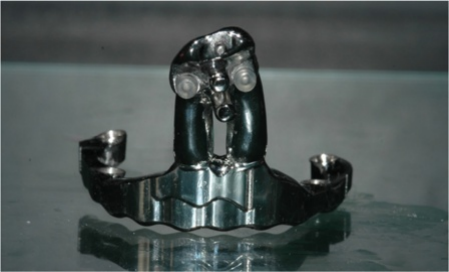
A front view of the bar with the intraoral portion and the metal extension for epithesis attachment

The patient received an implant-supported intra/extraoral rehabilitation with nasal epithesis and overdenture connected at the same metal framework due to the presence of an oronasal iatrogenic communication (Fig. 3). The nasal defect was classified into total (soft and hard tissues) rhinectomy. The palatal defect was localized at the premaxilla and was classified into “good” defect (resection margins into hard palate). Following the delivery of the prostheses, the patient showed satisfaction both for aesthetic and functional results and reverted to normal life achieving social integration (Fig. 4); he also reduced anxiolithic and antidepressive drug intake according to psychiatric counselling, and he is waiting to gradually stop them definitively. The patient did not receive radiotherapy and was non-smoker, two factors that are known to influence the success of implant therapy. He started an implant and prosthetic aftercare program.

### Discussion

Patients with advanced orofacial cancer may require extensive surgical resection; the wider and more evident is the amputated region, the more this condition is generating inability for patients [6]. Visible head site mutilation and functional impairment in speech prevent social reintegration, and abnormal self-perception leads patients to depression [6].

Even if modern surgery offers many techniques for reconstruction such as free flaps and rotation flaps, they are not indicated in all clinical cases. Because of the huge number of surgical sessions often required in reaching the wishing result, the use of local or microvascular flap could not be indicated in case of elderly patients or patients affected by cardiovascular or metabolic diseases. Moreover, a multistep surgical planning is not advisable in the absence of a complete sure compliance of the patient to the treatment [9]. Furthermore, recipient site complication can occur before and after harvesting or radiotherapy, when required, shall compromise the healing of the flap [9].

Nowadays, prosthetic extraoral rehabilitation is effective, less invasive because no additional surgical procedure is required, cosmetically satisfying and leads patients to a precocious social reintroduction. Additionally, intraoral restoration such as palatal obturator may allow speech and swallowing which play a crucial role in the retrieval of social life [8, 10].

Nasal defects are classified into partial, total and extended rhinectomy referred to soft tissue resection, bone and soft tissue amputation and bone and soft tissue associated to the maxilla or orbital excision [10].

Extraoral defects are usually restored by means of silicon epithesis; intraoral ones necessitate maxillary rehabilitation. In our case, since the premaxilla was lost, no implant insertion in the anterior region was possible. The importance of anterior implant anchorage is well documented even if a higher failure rate than the ones placed in the posterior maxilla is demonstrated [8, 10, 11].

In palatal cleft iatrogenic defects, implants insertion depends on bone residual amount, alveolar ridge height, radiotherapy and peri-implant soft tissue conditions [8, 10]. In patients who undergone radical surgery, all these requirements are often unfavourable and zygoma implants represent a valid alternative in offering prosthetic anchorage [2, 6, 10].

**Fig. 5:**
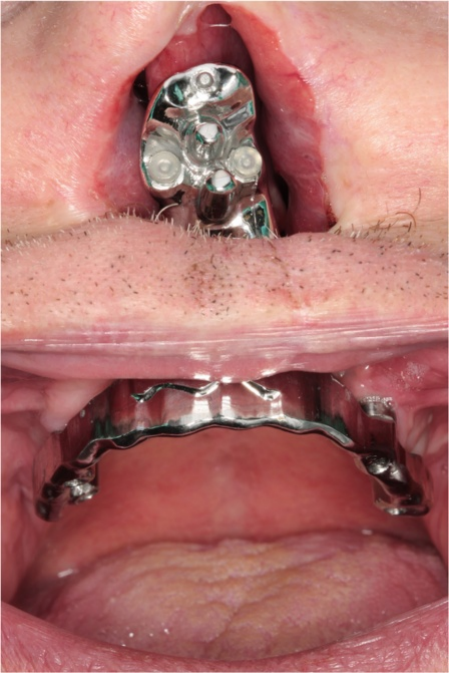
The intraoral bar crossing the palatal defect arising the nasal understructure

As far as prosthetic design is concerned, it is mandatory to avoid or, if not possible, limit as much as possible distal cantilever: given the absence of the premaxilla, an anterior cantilever is already present. Implant splintage is recommended [1, 8], and the bar design must respect technical data (implant-to-implant distance, cross-arch stabilization avoiding to cover oronasal communication and shape offering nasal epithesis connection) and clinical requirements (patient’s aftercare, visible inspection for follow-up). One of the most important technical issues is about oronasal communication: if the bar crosses, it is close to the upper lip, no obturator can be manufactured and the lack of vestibular seal may cause nasal flow during beverage swallowing (Fig. 5).

The combined zygoma-implant-supported prosthesis and nasal epithesis represents a new approach to rehabilitate wide complex midfacial defects. Nasal reconstruction, oroantral communication closure, labial competence correction and dental prosthetic rehabilitation are not commonly corrected by a unique surgical intervention or by a unique prosthetic rehabilitation. The prosthetic rehabilitation here presented allows to achieve all the above-mentioned goals by means of a single prosthesis.

Intraoral implants offer good anchorage for palatal obturator prosthesis, and extraoral implants’ use to support facial epithesis is well documented. Dawood describes a new implant design to support nasal epithesis and upper jaw prosthesis, but he reports just a single patient treatment [12]. Bowden reports zygoma implant placement horizontally below orbital floors and nasal prosthesis anchorage, but we managed with combined midfacial and palatal defects [2].

Prosthetic aftercare usually requires patient’s instruction about bar and implants’ daily hygienic procedures and silicone nasal epithesis cleaning [13, 14]. Despite careful home care, silicone facial prosthesis lifespan is 1.5/2 years on average because of discoloration, clip detachment from acrylic to silicone or acrylic carrier detachment to silicone, bad fit or silicone laceration [13, 14]. Unfavourable events for intraoral prosthesis are screw loosening and bar dislocation or screw fracture, obturator misfitting due to soft tissue remodelling, implant failure and prosthetic teeth fracture or excessive abrasion due to occlusal loss of balance [14].

Rethinking globally of the possible indications to the adoption of this technique and its advantages compared to reconstructive microsurgery, the use of zygoma-implant-supported prosthesis may be suitable for patients whose systemic conditions are poor. The duration of surgery and of the postoperative recovery would be remarkably shortened avoiding the complications related to the harvesting of a free flap. Closely related to this aspect, the cost-benefit ratio is definitely more convenient. This technique proves itself to be more easily manageable also in non-compliant patients or in patients with limited prognosis or high risk of recurrence, allowing the clinician a more effective inspection of the resected site during follow-up consults.

## Conclusions

**Fig. 6:**
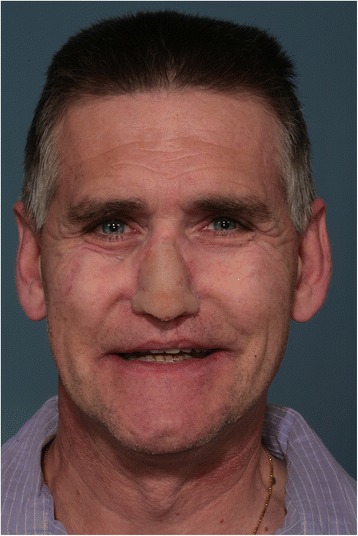
Frontal view of the patient after superior overdenture and nasal prosthesis delivery

**Fig. 7:**
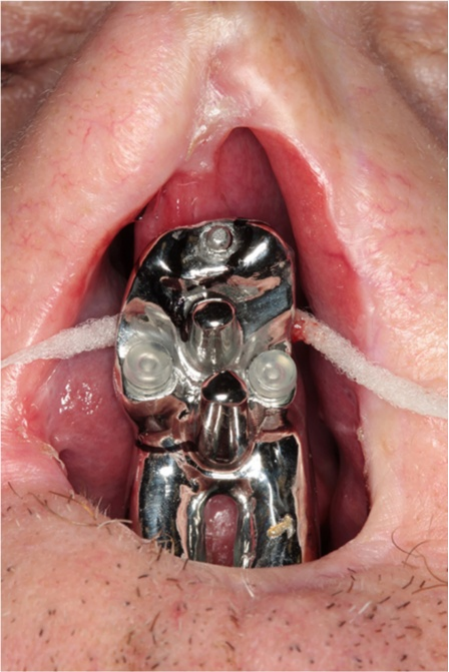
The epithesis allows both prompt inspection of the resection site and makes daily care easier

Implant-supported prosthesis is a valid method to restore resected oral and head cancer patients and offers a good chance to social reintegration. The aesthetic result and facial camouflage are more achievable by means of dentures and epithesis than with several reconstructive interventions. Furthermore, due to the high risk of recurrences, it is sometime mandatory to keep the defect inspectionable. Despite the average poor lifespan of prosthetic materials and the accurate professional and home care required by intraoral implants, prosthetic rehabilitation could be considered an effective and suitable method for rehabilitation of extensively resected head and neck cancer patients (Figs. 6 and 7).

## Consent

Written informed consent was obtained from the patient for publication of this case report and any accompanying images. A copy of the written consent is available for review by the Editor-in-Chief of this journal.
